# Supplementary material for: Expert and Interdisciplinary Analysis of AI-Driven Chatbots for Mental Health Support: Mixed Methods Study
Source: J Med Internet Res. 2025 Apr 25;27:e67114. doi: 10.2196/67114 (PMC12064976; doi:10.2196/67114)
Supplement: Multimedia Appendix 2 [file jmir_v27i1e67114_app2.pdf]

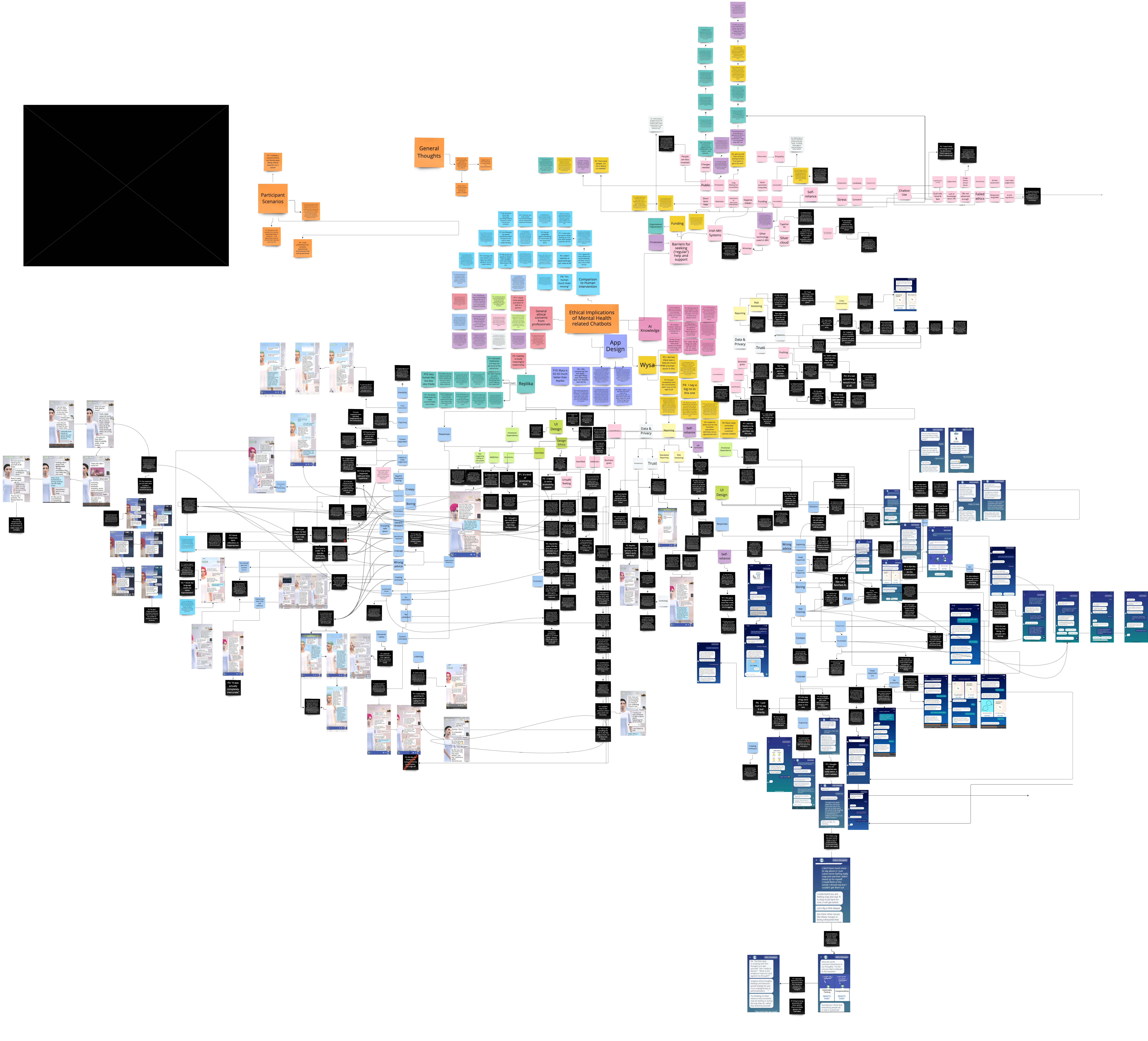

General Thoughts
Participant Scenarios
Ethical Implications of Mental Health related Chatbots
General ethical concerns from professionals
Comparison to Human intervention
AI Knowledge
App Design
Wysa
Replika
Barriers for seeking (regular) help and support
Irish MH Systems
Funding
Self-reliance
Chatbot Use
Stress
Failed ethics
Data & Privacy
Trust
UI Design
Design Ethics
Creepy
Boring
Bias
Silver cloud
